# Early posterior vault distraction osteogenesis changes the syndromic craniosynostosis treatment paradigm: long-term outcomes of a 23-year cohort study

**DOI:** 10.1007/s00381-024-06465-x

**Published:** 2024-06-21

**Authors:** Meagan Wu, Sarah L. Barnett, Benjamin B. Massenburg, Jinggang J. Ng, Dominic J. Romeo, Jesse A. Taylor, Scott P. Bartlett, Jordan W. Swanson

**Affiliations:** Division of Plastic, Reconstructive, and Oral Surgery, Children’s Hospital of Philadelphia, Pennsylvania, PA USA

**Keywords:** Craniosynostosis, Syndromic, Posterior vault distraction osteogenesis, Fronto-orbital advancement, Midface surgery

## Abstract

**Background:**

Early surgical management of syndromic craniosynostosis varies widely between centers, with patients typically undergoing multiple intracranial procedures through childhood. This study evaluates the long-term impact of early posterior vault distraction osteogenesis (PVDO) versus conventional treatment paradigms on the number and timing of subsequent craniofacial procedures.

**Methods:**

We retrospectively analyzed the longitudinal operative patterns of patients with syndromic craniosynostosis treated from 2000 to 2023 with greater than five years of follow-up. Outcomes of patients who underwent early PVDO and conventional vault reconstruction were compared.

**Results:**

Fifty-five patients met the inclusion criteria (30 PVDO and 25 conventional). Age at initial vault surgery was similar between the PVDO and conventional cohorts (7.6 vs. 8.8 months), as were baseline craniometrics (*p* > 0.05). Multiple fronto-orbital advancement (FOA) procedures were performed in only 1/30 (3%) PVDO-treated patient versus 12/25 (48%) conventionally-treated patients (*p* < 0.001). Twelve (40%) PVDO-treated patients did not undergo FOA at all. Among patients with Apert and Crouzon syndromes, fewer PVDO-treated patients required FOA prior to midface surgery (33% vs. 92%, *p* = 0.004) or repeat FOA (6% vs. 50%, *p* = 0.005) compared to conventionally-treated patients. Among patients with Muenke and Saethre–Chotzen syndromes, a similar proportion of patients required FOA in the PVDO and conventional cohorts (91% vs. 100%, *p* = 0.353), though no PVDO-treated patients required repeat FOA (0% vs. 44%, *p* = 0.018).

**Conclusions:**

Early PVDO is associated with a reduction in the high burden of both FOA and, critically, revisionary frontal procedures which are commonly seen in conventionally-treated patients with syndromic craniosynostosis.

**Supplementary Information:**

The online version contains supplementary material available at 10.1007/s00381-024-06465-x.

## Introduction

The surgical management of syndromic craniosynostosis is challenging due to complex craniofacial dysmorphology and cranio-cerebral disproportion [1], with the choice of technique and timing varying widely between institutions [2–5]. Three intertwined goals of cranial vault remodeling for multi-suture craniosynostosis are to (1) prevent or treat elevated intracranial pressure (ICP), (2) normalize calvarial shape, and (3) minimize the cumulative burden of craniofacial surgical intervention [6]. As the turribrachycephaly in these patients involves both frontal retrusion and occipital flattening, surgeons may choose to address the posterior before anterior vault, provided there is sufficient globe protection [7].

Over the last decade, posterior vault distraction osteogenesis (PVDO) has become an increasingly favored option for early management. Since 2009, our institution has employed PVDO as the first-line intervention for most syndromic patients, typically at six to nine months of age [2]. An earlier decompressive craniectomy may be performed if signs of elevated ICP are present. Among its advantages, PVDO provides greater volume expansion than fronto-orbital advancement (FOA) [8–17] and has a favorable safety profile [7, 11, 18–22]. Postoperative improvements in cranial morphology have also been established, including increased posterior cranial height [11], increased posterior cranial base length and foramen magnum size [23], and decreased frontal bossing [11, 16].

Perhaps one of the greatest potential benefits of PVDO over conventional treatment paradigms lies in the reduced future surgical burden resulting from a larger, more gradual, and less devascularizing early vault expansion [2]. We previously described our initial six-year experience with PVDO as the first-line intervention for syndromic craniosynostosis and found that early PVDO was associated with later and fewer FOAs in the short term compared to conventional remodeling [2]. As this PVDO-treated cohort continues to age, many patients have now grown through the period of midface surgery, presenting the opportunity to longitudinally assess the operative courses of our syndromic population from initial vault expansion through frontofacial advancement. By analyzing the number and timing of operations, this long-term study compares our 15-year experience with a PVDO-based protocol to a conventional protocol. We hypothesized that early PVDO would be associated with a reduced burden of craniofacial surgical intervention, including the rate of revisionary frontal procedures, with syndrome-specific variations.

## Methods

### Study population

Following Institutional Review Board approval at the Children’s Hospital of Philadelphia, a retrospective review was performed of patients with a diagnosis of Apert, Crouzon, Pfeiffer, Muenke, or Saethre-Chotzen syndrome treated from 2000 to 2023. Patients were included if they had a minimum follow-up of five years and presented under the age of 2.5 years. Cohorts were divided into those who underwent early PVDO (2009 onwards) or conventional vault reconstruction (before 2009) without PVDO to compare the number and timing of operations between treatment groups. Patients who underwent prior vault surgery before PVDO were excluded. All craniofacial interventions were recorded, including vault procedures, frontofacial procedures, and ventriculoperitoneal (VP) shunt placements and revisions. Preoperative computed tomography (CT) scans were used to evaluate for underlying differences in clinical severity.

### Surgical technique

**Fig. 1:**
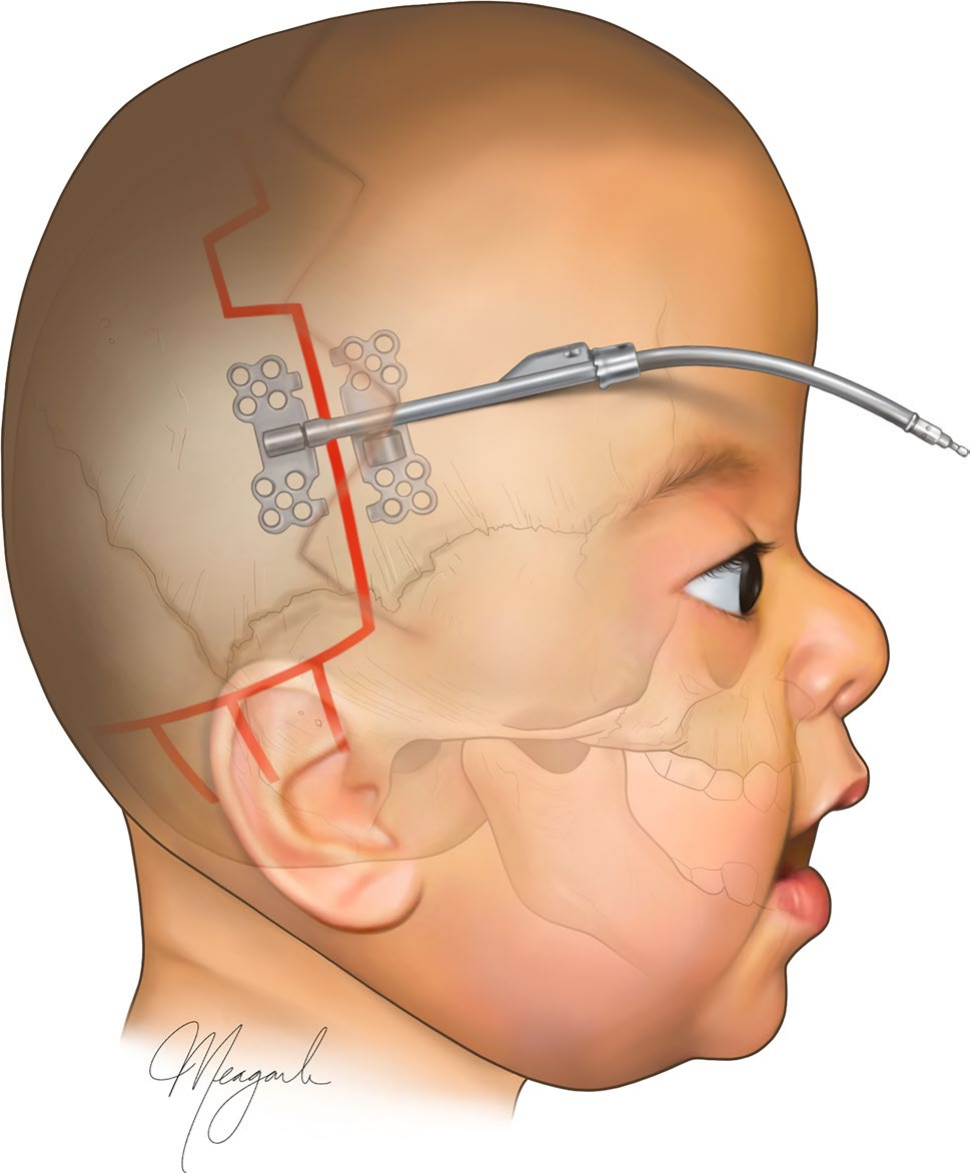
Illustration of PVDO depicting the coronal skin incision, osteotomy markings with barrel staving, and internal distraction device

Our surgical approach for PVDO has been previously described [7]. With the patient lying prone, the posterior vault is approached via a bicoronal incision. At the occipital bone inferior to the distraction segment, barrel stave osteotomies are made, green-sticked, and outfractured posteriorly. Bilateral 1.5-mm cranial distractors with 30- to 40-mm barrels are placed in a colinear, parasagittal fashion with a posteroinferior vector of distraction (Fig. 1). For patients with normo- or scaphocephaly, a transverse vector with distractors placed perpendicular to a midline sagittal craniotomy may be utilized [24]. Distraction begins after a 2-day latency period at 1 mm/day and continues until slight overcorrection of the brachycephaly. The consolidation period lasts two to three months. Our conventional approach was primarily FOA, which expands the cranial vault and advances the supraorbital bar to improve ocular protection and frontal morphology [25]. A small number of patients alternatively underwent posterior vault reconstruction (PVR). Both techniques have been described in detail [25, 26].

Operations were performed by three pediatric craniofacial surgeons, and any differences in outcomes based on treating surgeon were analyzed. The decision-making of type and timing of primary and secondary procedures in both cohorts was informed by consensus input from the institutional multi-disciplinary craniofacial team including neurosurgical, neuroophthalmological, otolaryngological, and psychosocial expertise. The criteria for FOA centered on the presence of anterior cerebral volume restriction, frontal craniofacial dysmorphology, and/or ocular exposure for which direct manipulation was thought superior to alternate cranial vault interventions with the opportunity for passive correction with growth and alleviation of pathophysiologic brain-directed secondary growth.

### Craniometrics

Preoperative fine-cut (1 mm or less) head CT scans were analyzed on Mimics Version 23.0 (Materialise, Leuven, Belgium) to evaluate baseline morphometrics. Five linear distances [27], frontal bossing angle [11, 28], and turricephaly index [29, 30] were measured. Distances included cranial width, cranial length, and three cranial heights (anterior, middle, and posterior) (Supplemental Fig. 1). The Frankfort Horizontal (FH) plane was established, perpendicular to which a midsagittal plane intersecting the sella and nasion was established. Cranial width was measured from the inner cortex of one euryon to the contralateral euryon in a line parallel to the FH plane. In the midsagittal plane, cranial length was measured from the nasion to the inner cortex of the opisthocranion; anterior, middle, and posterior cranial heights were measured from the nasion, sella, and basion, respectively, to the inner cortex of the calvarium in a line perpendicular to a line connecting the sella and nasion (SN). Frontal bossing angle was measured between the SN line and a line connecting the nasion to the most anterior portion of the frontal bone (B point). Turricephaly index was calculated as the ratio of the maximal fronto-occipital length, as measured by a line from the B point to the opisthocranion, over middle cranial height.

### Statistical analysis

Data were analyzed using R 4.3.1 (R Foundation for Statistical Computing, Vienna, Austria). Demographic and surgical data between treatment cohorts were compared using the chi-square test. Univariate analysis was conducted using the chi-square and Fisher’s exact tests for categorical variables and the Mann–Whitney U test for continuous variables. Kaplan–Meier analysis evaluated the impact of PVDO on time to FOA and midface surgery. Craniometrics were compared between treatment cohorts using the Mann–Whitney U test and between syndrome groups using the Kruskal–Wallis test. For syndrome-specific comparisons by treatment protocol, exact age thresholds were determined by cohort composition. Statistical significance was set at *p* < 0.05.

## Results

### Operative patterns

**Fig. 2:**
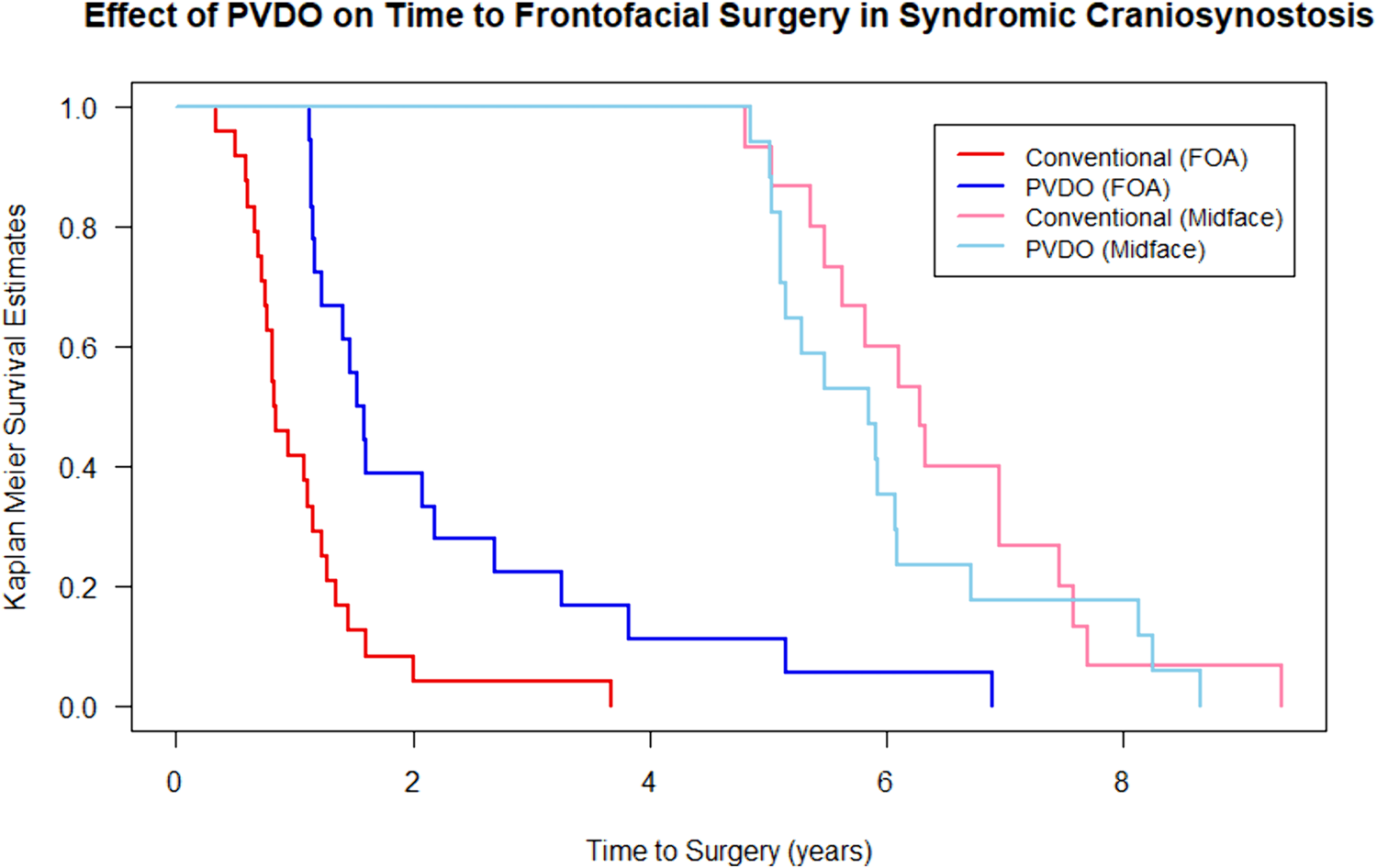
Kaplan–Meier analysis demonstrated a delay to first FOA (red and blue lines) among patients in the PVDO cohort compared to conventional cohort (*p* < 0.001), with no significant difference in time to first midface surgery (pink and light blue lines) between cohorts (*p* = 0.400). *PVDO*, posterior vault distraction osteogenesis; *FOA*, fronto-orbital advancement

Of 131 syndromic patients treated from 2000 to 2023, 55 met inclusion criteria: 30 (55%) underwent early PVDO and 25 (45%) underwent conventional reconstruction. Median age at presentation was similar between the PVDO and conventional cohorts (1.3 vs. 1.8 months), as was median age at initial surgery (7.6 vs. 8.8 months, Table 1). Primary FOAs were delayed to an older mean age in the PVDO versus conventional cohort (2.3 ± 1.6 vs. 1.1 ± 0.7 years; *p* < 0.001; Table 2 and Fig. 2). In the PVDO cohort, 12/30 (40%) patients did not require any FOA procedures  at greater than age six, with 8/12 (67%) of these patients undergoing monobloc advancement at a mean age of 5.7 ± 1.1 years. Multiple FOAs were performed in only 1/30 (3%) PVDO-treated patient compared to 12/25 (48%) conventionally-treated patients (*p* < 0.001). Patients underwent a lower mean number of FOAs in the PVDO cohort (0.6 ± 0.6 vs. 1.4 ± 0.6 surgeries; *p* < 0.001) and a similar mean number of total craniofacial interventions (2.9 ± 1.1 vs. 3.2 ± 2.3 surgeries; *p* = 0.741) compared to the conventional cohort. The rate of VP shunt placements and revisions was similar between cohorts **(**Table 2**)**. The number of FOAs, both primary and secondary, as well as total craniofacial interventions did not differ based on the treating surgeon (*p* > 0.05). All findings remained true when analyzing interventions in the first eight years of life in a subgroup of 46 patients presenting before age one (Supplemental Table 1).

**Table 1:** Demographic characteristics of the PVDO and conventional cohorts (*n* = 55)

	**PVDO cohort (%)**	**Conventional Cohort (%)**	***p***
No. of patients	30 (55)	25 (45)	
Sex			
Male	11 (37)	11 (44)	0.580
Female	19 (63)	14 (56)	
Age at presentation, yr			
Mean ± SD	0.4 ± 0.6	0.5 ± 0.6	0.559
Median [IQR]	0.1 [0.0, 1.0]	0.2 [0.0, 0.9]	
Age at initial vault surgery, yr			
Mean ± SD	1.0 ± 0.8	1.2 ± 1.8	0.754
Median [IQR]	0.6 [0.5, 1.3]	0.7 [0.5, 1.0]	
Postoperative follow-up, yr			
Mean ± SD	9.0 ± 2.6	14.3 ± 5.3	**< 0.001**
Median [(IQR]	8.9 [6.9, 11.2]	15.4 [10.9, 18.0]	
Syndrome type			
Apert	7 (23)	8 (32)	0.424
Crouzon	10 (32)	6 (24)	
Pfeiffer	2 (7)	2 (8)	
Muenke	5 (16)	6 (24)	
Saethre–**­­­**Chotzen	6 (19)	3 (12)	
No. of patients with Chiari malformation	4 (13)	3 (12)	0.883

Bolded *p*-value indicates statistical significance

*PVDO* posterior vault distraction osteogenesis, *SD* standard deviation, *IQR* interquartile range

**Table 2:** Surgical interventions in the PVDO and conventional cohorts (*n* = 55)

	**PVDO cohort (%)**	**Conventional cohort (%)**	***p***
No. of patients	30 (55)	25 (45)	
Mean age at primary FOA, yr	2.3 ± 1.6	1.1 ± 0.7	**< 0.001**
Mean age at secondary FOA, yr	8.7	6.5 ± 3.2	
No. of patients requiring secondary FOA	1 (6)	12 (48)	**0.005**
No. of FOAs	0.6 ± 0.6	1.4 ± 0.6	**< 0.001**
0	12 (40)	1 (4)	
1	17 (57)	12 (48)	
2	1 (3)	12 (48)	
Mean age at first midface surgery, yr	6.0 ± 1.2	7.2 ± 3.2	0.155
Transcranial	5.7 ± 1.0	6.0 ± 0.6	0.161
Subcranial	6.5 ± 1.3	8.3 ± 4.1*	0.354
No. of patients requiring midface surgery	16 (57)	16 (64)	0.580
Transcranial	8 (27)	11 (44)	0.208
Subcranial	9 (30)	5 (20)	0.208
No. of PVDOs			
1	29 (97)	—	
2	1 (3)	—	
Mean no. of major craniofacial interventions	2.9 ± 1.1	3.2 ± 2.3	0.741
1	1 (3)	7 (28)	
2	12 (40)	4 (16)	
3	10 (33)	7 (28)	
4	5 (17)	2 (8)	
5	1 (3)	2 (8)	
6	1 (3)	0 (0)	
7	0 (0)	1 (4)	
8	0 (0)	1 (4)	
9	0 (0)	0 (0)	
10	0 (0)	1 (4)	
No. of patients requiring VP shunts	7 (23)	3 (12)	0.278
Mean age at shunt placement, yr	0.8 ± 0.8	0.9 ± 0.4	0.429
Mean no. of shunt revisions	2.5 ± 2.8	1.3 ± 1.5	0.637

Bolded *p*-value indicates statistical significance

*PVDO* posterior vault distraction osteogenesis, *FOA* fronto-orbital advancement, *VP* ventriculoperitoneal

*7.4 ± 2.8 year after exclusion of one patient who underwent Le Fort I advancement at age 18.2

### Syndrome-specific outcomes

**Fig. 3:**
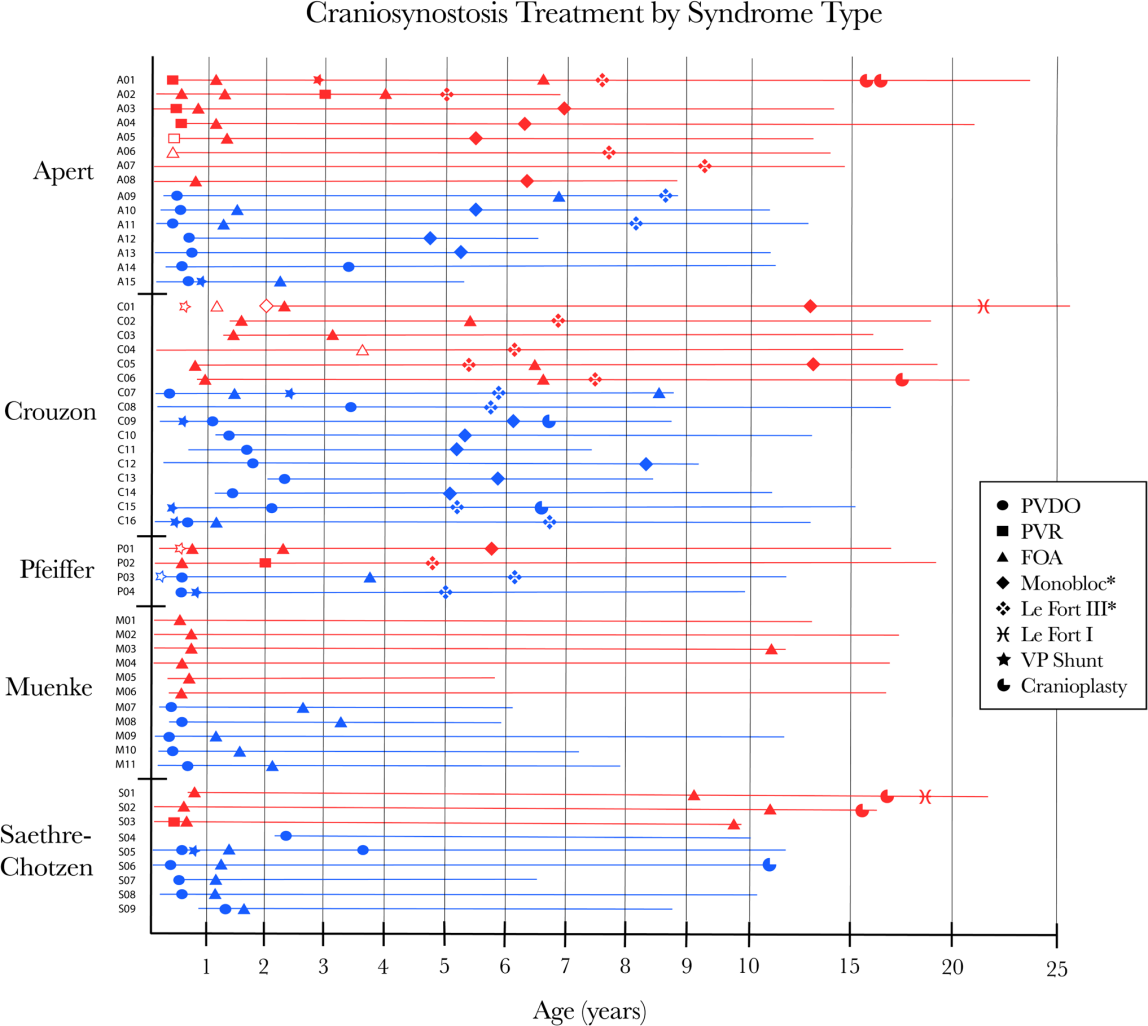
Surgical treatment of craniosynostosis in patients with Apert, Crouzon, Pfeiffer, Muenke, and Saethre–Chotzen syndromes. Icons represent different surgical procedures (*filled icons*, performed at our institution; *open icons*, performed at another institution). Lines represent the period of patient care at our institution. Patients in the conventional cohort (red) typically undergo initial treatment with FOA (triangle) with a small portion undergoing PVR (square), compared to patients in the PVDO cohort (blue) who undergo initial treatment with PVDO (square). *PVDO*, posterior vault distraction osteogenesis; *PVR*, posterior vault reconstruction; *FOA*, fronto-orbital advancement; *VP*, ventriculoperitoneal

Baseline craniometrics were similar between treatment cohorts and varied by syndrome type (Supplemental Tables 2 and 3). The frontal bossing angle was largest in the Apert, Muenke, and Saethre–Chotzen groups at 129° ± 8.3°, 125.6° ± 2.9°, and 124.8° ± 7.3°, respectively (*p* = 0.009). Turricephaly index was smallest, indicating greater turricephaly, in Apert and Saethre–Chotzen syndromes at 1.17 ± 0.13 and 1.17 ± 0.08, respectively, followed by Muenke syndrome at 1.25 ± 0.07 (*p* = 0.007). Importantly, number and timing of operations varied by syndrome type, as detailed below (Fig. 3).

#### Apert syndrome

Of patients with Apert syndrome who underwent vault expansion and have reached age six (*n* = 13), only 3/6 (50%) PVDO-treated patients underwent FOA compared to 7/7 (100%) conventionally-treated patients (*p* = 0.033), of which 2/7 (29%) required repeat FOA. A similar number of patients underwent transcranial (50% vs. 57%) and subcranial (33% vs. 43%) midface procedures in the PVDO and conventional cohorts (*p* > 0.05). Patients underwent a similar number of craniofacial interventions in the PVDO and conventional cohorts (3.0 ± 1.0 vs. 3.3 ± 1.9 surgeries, *p* = 1.000).

#### Crouzon syndrome

Of patients with Crouzon syndrome who have reached age seven (*n* = 16), 2/10 (20%) PVDO-treated patients underwent FOA compared to 6/6 (100%) conventionally-treated patients (*p* = 0.006), of which 5/6 (83%) required repeat FOA. While more PVDO-treated patients underwent transcranial than subcranial midface procedures (60% vs. 20%, *p* = 0.143), one conventionally-treated patient underwent both Le Fort III and monobloc advancements at age 5.4 and 13.4 years, respectively, after FOA. Patients underwent fewer total craniofacial interventions in the PVDO compared to conventional cohort (3.0 ± 0.8 vs. 4.8 ± 3.5 surgeries, *p* = 0.001).

#### Pfeiffer syndrome

Patients with Pfeiffer syndrome who have reached age ten (*n* = 4) displayed a similar pattern as patient with Apert and Crouzon syndromes, as 1/2 (50%) PVDO-treated patients underwent FOA compared to both (100%) conventionally-treated patients (*p* = 0.248), both of whom required a secondary FOA or PVR. Both (100%) PVDO-treated patients underwent subcranial midface procedures, whereas 1/2 (50%) conventionally-treated patient required a monobloc advancement after two FOAs. Patients in both treatment cohorts underwent an average of 3.5 ± 0.7 craniofacial interventions.

#### Muenke syndrome

All patients with Muenke syndrome who have reached age six (*n* = 11) underwent FOA regardless of treatment protocol, with 1/6 (17%) conventionally-treated patient requiring repeat FOA at age 11.4 years. No patients required midface surgery. Patients underwent a greater mean number of craniofacial interventions in the PVDO versus conventional cohort (2.0 ± 0.0 vs. 1.2 ± 0.4 surgeries, *p* = 0.012).

#### Saethre–Chotzen syndrome

Among patients with Saethre–Chotzen syndrome who have reached age six, 5/6 (83%) PVDO-treated patients underwent FOA compared to 3/3 (100%) conventionally-treated patients (*p* = 0.453), all three of whom required repeat FOA after age nine. No patients underwent midface surgery excluding one conventionally-treated patient who underwent Le Fort I advancement in adulthood. Patients underwent a similar number of craniofacial interventions in the PVDO and conventional cohorts (3.0 ± 1.8 vs. 3.3 ± 0.6 surgeries, *p* = 0.596).

Finally, we evaluated patients with Apert/Crouzon and Muenke/Saethre–Chotzen syndromes as two combined groups. In the Apert/Crouzon group (*n* = 31), fewer patients required FOA prior to midface surgery (33% vs. 92%, *p* = 0.003) or repeat FOA (6% vs. 50%, *p* = 0.005) in the PVDO versus conventional cohort. In the Muenke/Saethre–Chotzen group (*n* = 20), the proportion of patients undergoing FOA did not differ between the PVDO and conventional cohorts (91% vs. 100%, *p* = 0.353), though no PVDO-treated patients required repeat FOA (0% vs. 44%, *p* = 0.018).

## Discussion

**Fig. 4:**
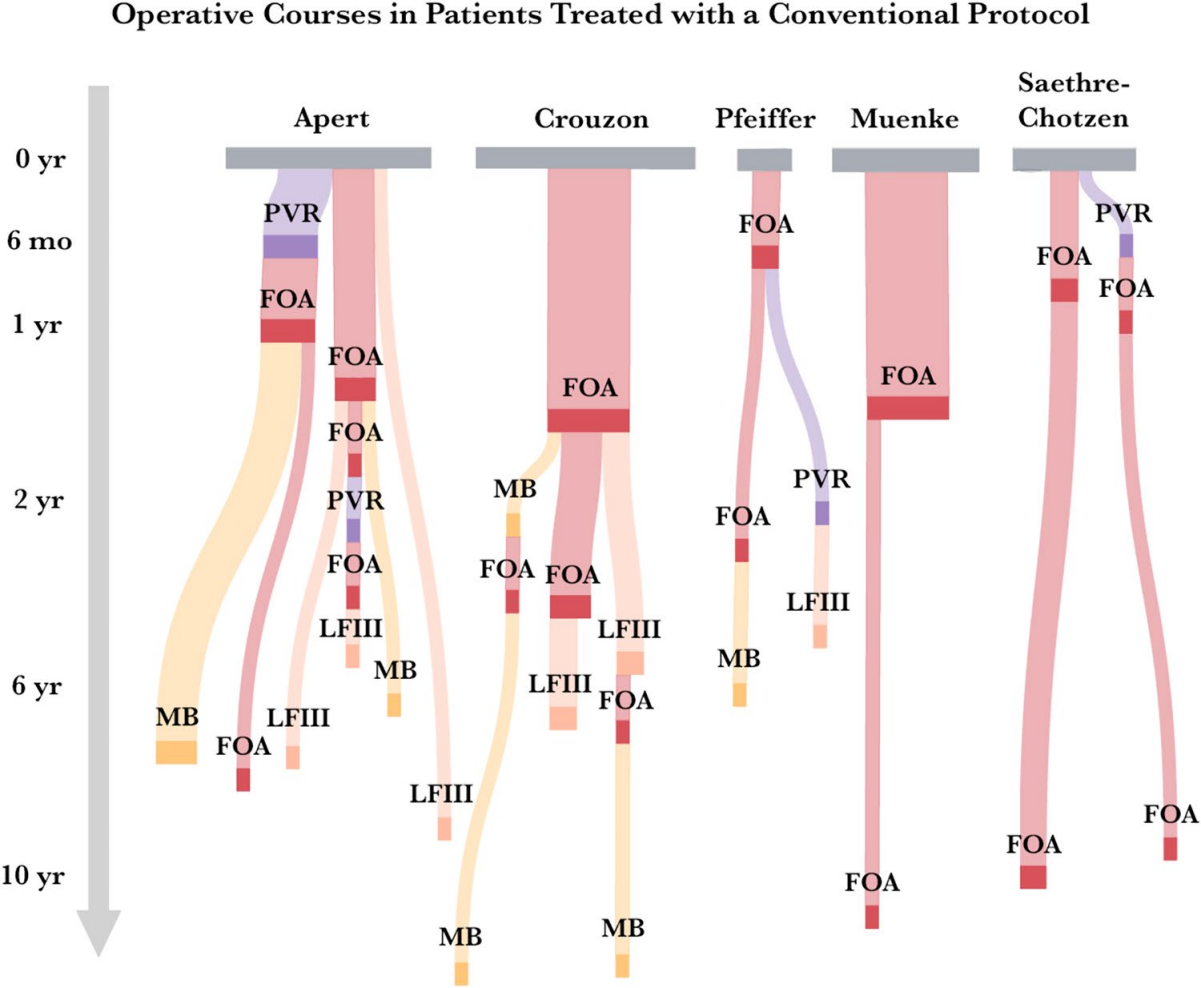
Syndrome-specific operative courses in patients treated with a conventional protocol, with node size and flow width representing the relative proportion of patients undergoing that particular care pathway. *PVDO*, posterior vault distraction osteogenesis; *FOA*, fronto-orbital advancement; *MB*, monobloc advancement; *LFIII*, Le Fort III advancement

**Fig. 5:**
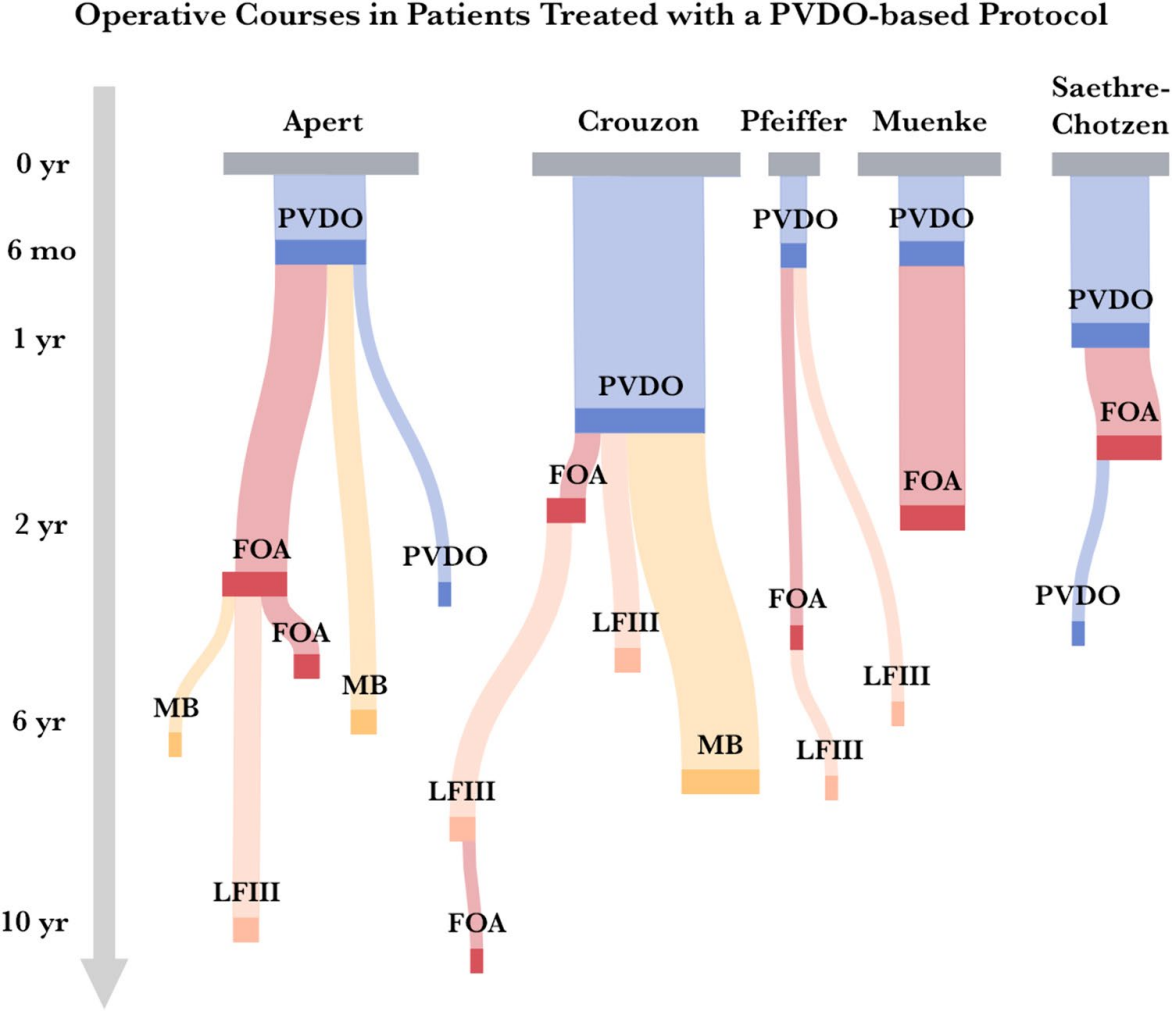
Syndrome-specific operative courses in patients treated with a PVDO-based protocol, with node size and flow width representing the relative proportion of patients undergoing that particular care pathway. *PVDO*, posterior vault distraction osteogenesis; *FOA*, fronto-orbital advancement; *MB*, monobloc advancement; *LFIII*, Le Fort III advancement

With the adoption of early PVDO, an important paradigm shift has been observed over the last 15 years, and several key findings emerged upon analyzing craniofacial treatment patterns through elementary age in comparison to the conventional protocol. First, the conventional protocol relied almost exclusively on FOA for initial vault expansion, and half of these patients required secondary or tertiary FOA due to relapse or pseudorelapse (Fig. 4). Second, while the PVDO-based protocol was not associated with fewer craniofacial operations performed overall, it has effectively re-distributed cranial expansion from the anterior to the whole skull. Frontal surgery has therefore been increasingly performed on an as-needed basis and can be combined with midface surgery at an older age in many cases as a single-stage frontofacial advancement. Third, while the PVDO-based protocol showed a decreased need for secondary FOA across all syndrome groups, pathway patterns for specific syndromes have emerged. Among patients with Apert and Crouzon syndromes, it has enabled passive frontofacial remodeling from posterior vault expansion, reducing the absolute need for frontal correction; on the other hand, it has been associated with a higher volume of surgical procedures among patients with Muenke syndrome in the process of pursuing posterior expansion prior to frontal normalization (Fig. 5).

**Fig. 6:**
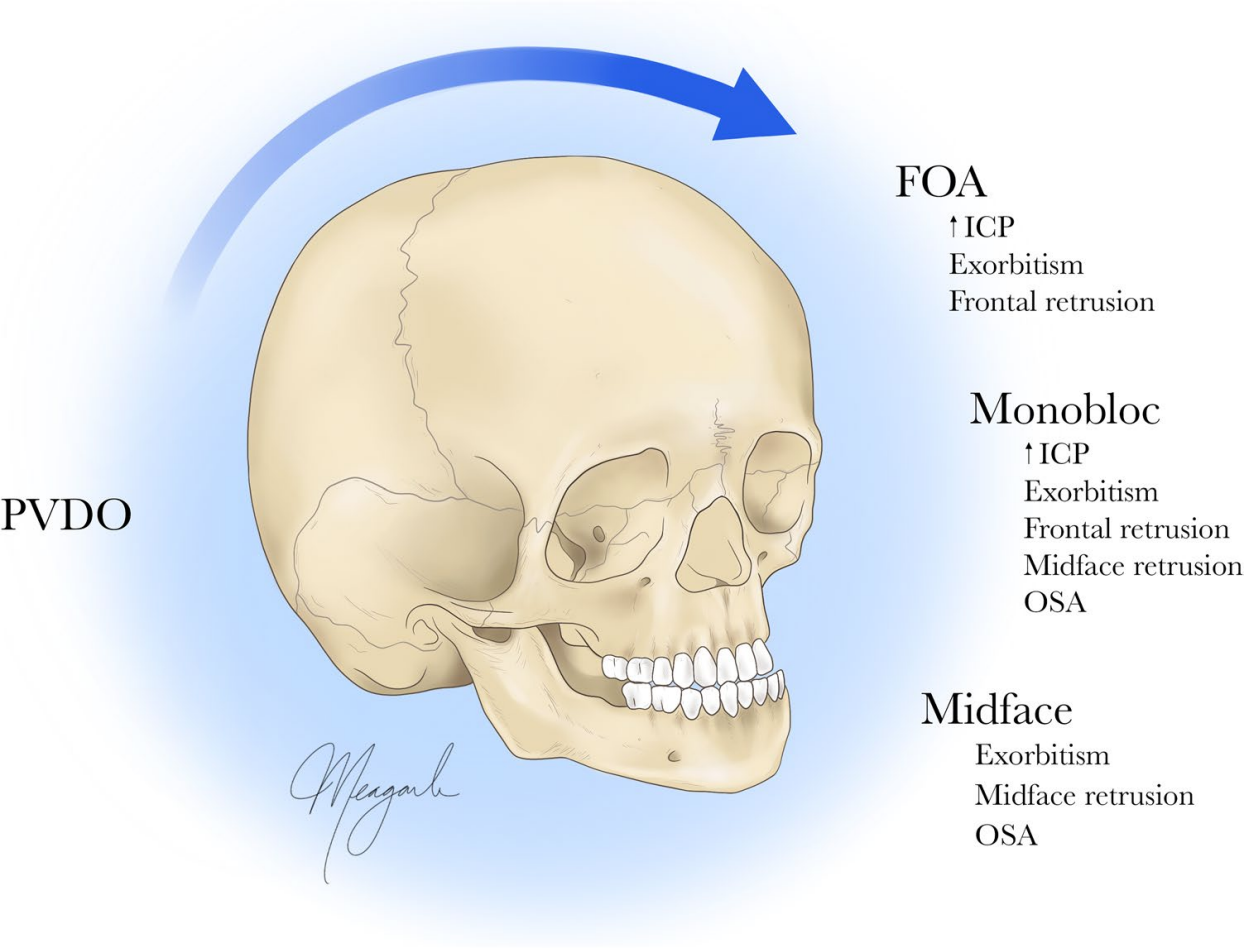
Schematic illustrating our institution’s treatment algorithm based on early PVDO followed by frontofacial interventions, as indicated, for the treatment of syndromic craniosynostosis. *PVDO*, posterior vault distraction osteogenesis; *FOA*, fronto-orbital advancement; *ICP*, intracranial pressure; *OSA*, obstructive sleep apnea

Our data show that early PVDO is associated with a reduced rate of secondary FOAs, affirming that the benefits of early PVDO on frontal morphology and future surgical burden extend beyond the primary frontal procedure. In our series, 12 (40%) patients did not require FOA at all after PVDO. Four of these patients experienced passive anterior cranial normalization, with two undergoing LFIII advancement at ages five and six and the other two, currently ages ten and 12, only awaiting orthognathic surgery. The other eight patients underwent monobloc advancement at an average age of 5.7 ± 1.1 years; as such, they were able to sustain orbital growth to skeletal maturity and undergo transcranial frontofacial advancement as their second-stage procedure, avoiding a three-stage approach entirely (Fig. 6). This has important implications, as complication rates rise with each subsequent approach to the anterior cranium [2, 31]. Wagner et al. [32] found that in syndromic patients undergoing monobloc advancement at our institution, prior FOA was associated with increased major complications and dural tears. Therefore, in the absence of ICP concerns and severe exorbitism, advancement of the supraorbital bar via FOA may be achieved through monobloc advancement at a later age. Patient morbidity is thus reduced, both directly due to the technical advantages of PVDO and indirectly through the improved safety of subsequent procedures.

Upon further analysis, we found that treatment patterns varied by syndrome type, affirming that early PVDO has differential outcomes depending on phenotype. Early PVDO was associated with a reduced number of FOAs and total craniofacial operations in patients with Apert, Crouzon, and Pfeiffer syndromes, having mitigated the need for both primary and secondary FOAs to address relapse and pseudorelapse. In contrast, the PVDO-based protocol was associated with a greater number of craniofacial procedures overall in patients with Muenke syndrome, with surgeries per patient increasing from one to two in the era of PVDO. Similarly, all but one patient with Saethre–Chotzen syndrome continued to require FOA after PVDO.

**Fig. 7:**
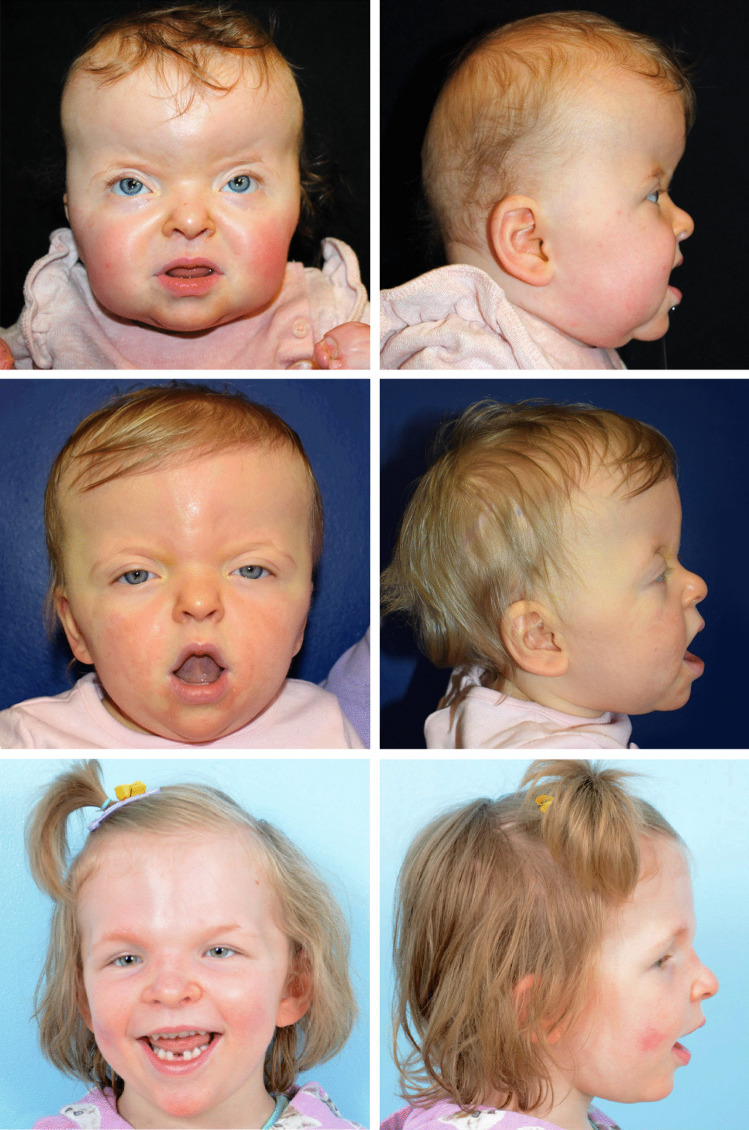
A female patient with Apert syndrome (A12) who underwent PVDO at nine months of age is shown preoperatively (top row) and postoperatively at 16 months of age (middle row). She underwent monobloc advancement at age six and is shown postoperatively at age 11 (bottom row). She has had no other craniofacial procedures

**Fig. 8:**
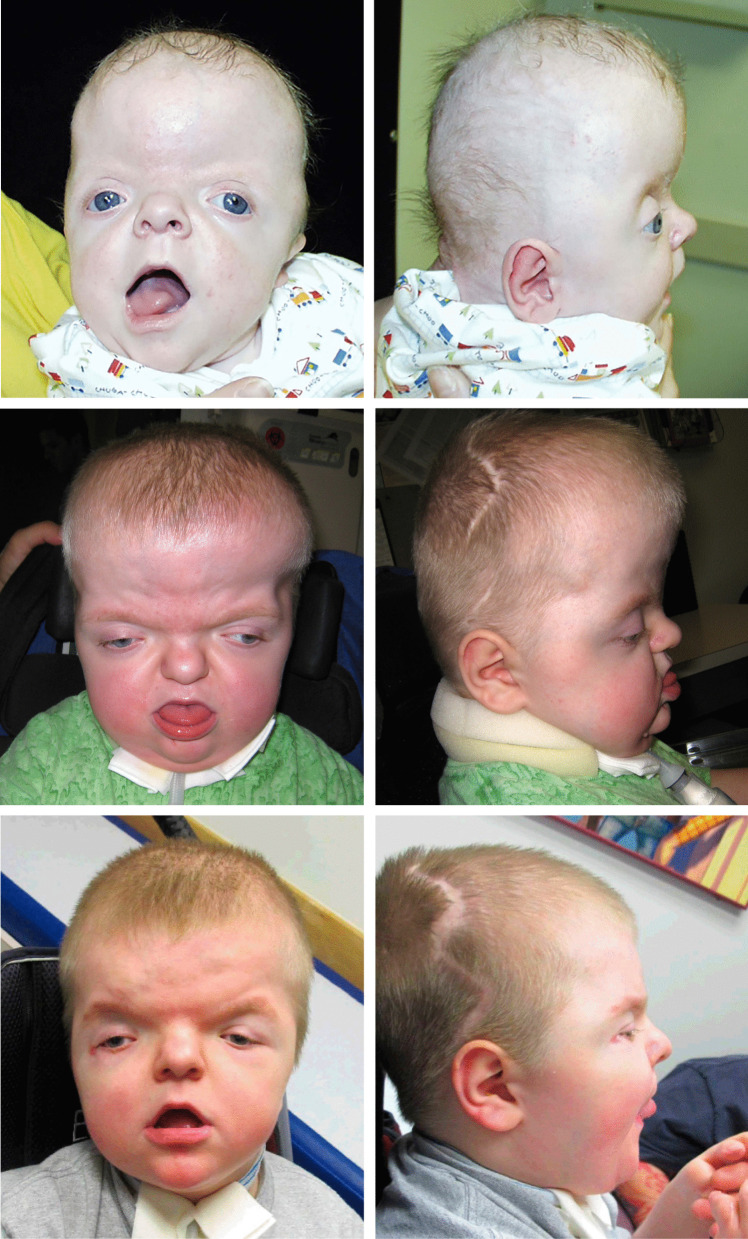
A male patient with Apert syndrome (A02) who underwent initial FOA at six months of age is shown preoperatively (top row) and postoperatively at age 2.5 years (middle row). He subsequently underwent two revisionary FOAs and one PVR procedure prior to undergoing Le Fort III midface advancement at age six and is shown postoperatively at age 12 (bottom row)

Such syndrome-specific patterns urge us to reconsider how our institution's current PVDO-based algorithm differentially benefits certain populations, as the syndromic craniosynostoses involve a spectrum of calvarial and periorbital dysmorphology, midface hypoplasia, and functional impairment [1]. Apert syndrome, for instance, carries a greater risk of developmental delay [33]. Several studies have also reported higher reoperation rates for frontal retrusion after a primary FOA in patients with Apert syndrome [34–36]. The Erasmus unit argues for treating patients with Apert, Crouzon, and Pfeiffer syndromes with a back-first approach because the larger volume of expansion better addresses intracranial hypertension (ICH), as supported by a greater reduction in incidence of papilledema and tonsillar herniation after occipital expansion versus FOA [37]. These findings underscore the need for robust intracranial expansion, as provided by PVDO, in Apert and Crouzon syndromes. The benefits of early PVDO can be seen when comparing several cases in our series with differing operative courses. A patient with Apert syndrome and another with Crouzon syndrome both underwent early PVDO and did not require additional surgery until their monobloc advancements at ages five and eight, respectively (Fig. 7 and Supplemental Fig. 2). In contrast, a conventionally-treated patient with Apert syndrome underwent FOA and required two revisionary FOAs and one PVR procedure before undergoing LFIII advancement at age six (Fig. 8).

On the other hand, FOA may be more advantageous as the primary reshaping procedure for Muenke craniosynostosis to address isolated frontal bossing, turricephaly, and less severe cranial dysmorphology [38, 39]. This is supported by the relatively greater frontal bossing angle, greater anterior cranial height, and lower Turricephaly Index in our Muenke and Saethre–Chotzen groups at baseline upon craniometric analysis. Our findings are consistent with those of the Erasmus team, who treats patients with Muenke syndrome with a single-stage FOA at six to nine months of age [40]. Moreover, their Muenke cohort had only a 3–8% risk of developing ICH and a low prevalence of obstructive sleep apnea, hydrocephalus, and venous outflow obstruction [40]. Similarly, a single-stage FOA was sufficient to prevent ICH in 80% of their patients with Saethre–Chotzen syndrome [41]. Because passive correction did not obviate the need for frontal surgery in our Muenke group (Fig. 5), further investigation of morphometric and/or neurocognitive outcomes is required to assess the potential utility, or lack thereof, of earlier and more robust vault expansion in this syndrome group. While almost all PVDO-treated patient with Saethre–Chotzen syndrome similarly required a subsequent FOA, no secondary FOAs have been observed after PVDO (Fig. 5), whereas all conventionally-treated patients have required a secondary FOA due to relapse or pseudorelapse (Fig. 4). This favorable difference in our Saethre–Chotzen group may be due to more global cranial expansion achieved via a combined back-front approach and/or older age at time of FOA. In the absence of data suggesting the superiority of repeat FOAs in this syndrome group, we see advantage in what appears to be definitive correction of craniofacial dysmorphology at a young age via a PVDO-based approach.

This study has several shortcomings. Comparison of cranial expansion techniques at a single institution is challenging, as operations are not performed in isolation but rather as a part of protocolized care. There was also variability in timing of presentation and follow-up. Of note, PVDO was consistently performed around six months of age in Apert, Pfeiffer, and Muenke syndromes but from three months up through two years of age in Crouzon and Saethre–Chotzen syndromes, even for patients followed from infancy. This discrepancy may reflect less concern for elevated ICP in certain patient groups. Nonetheless, our assessment of preoperative craniometric parameters validates the comparability between treatment cohorts, while our subgroup analysis of patients optimally presenting before age one corroborates our conclusions. While other centers have advocated for PVDO as the primary intervention for syndromic craniosynostosis with favorable outcomes [4, 5, 30, 42, 43], none have provided follow-up beyond a few years postoperatively.

## Conclusions

This long-term cohort study highlights an important shift in the syndromic craniosynostosis treatment paradigm with the implementation of PVDO, which has the potential to delay and alleviate the need for secondary frontal procedures. Granted that patients present in a timely fashion, total surgical burden is reduced. As variations in treatment patterns by syndrome type became increasingly apparent during the period of mixed dentition and through skeletal maturity, our PVDO-based algorithm should be further tailored to the functional and aesthetic goals of each patient.

### Supplementary Information

Below is the link to the electronic supplementary material.

Supplementary file1 (TIF 28700 KB)

Supplementary file2 (PNG 4280 KB)

Supplementary file3 (DOCX 14.6 KB)

Supplementary file4 (DOCX 13.8 KB)

Supplementary file5 (DOCX 14.6 KB)

## Data Availability

The data discussed in this manuscript is available upon request from the corresponding author.
