# Supplementary figures and images for: Early posterior vault distraction osteogenesis changes the syndromic craniosynostosis treatment paradigm: long-term outcomes of a 23-year cohort study
Source: Childs Nerv Syst. 2024 Jun 21;40(9):2811–23. doi: 10.1007/s00381-024-06465-x (PMC11322207; doi:10.1007/s00381-024-06465-x)

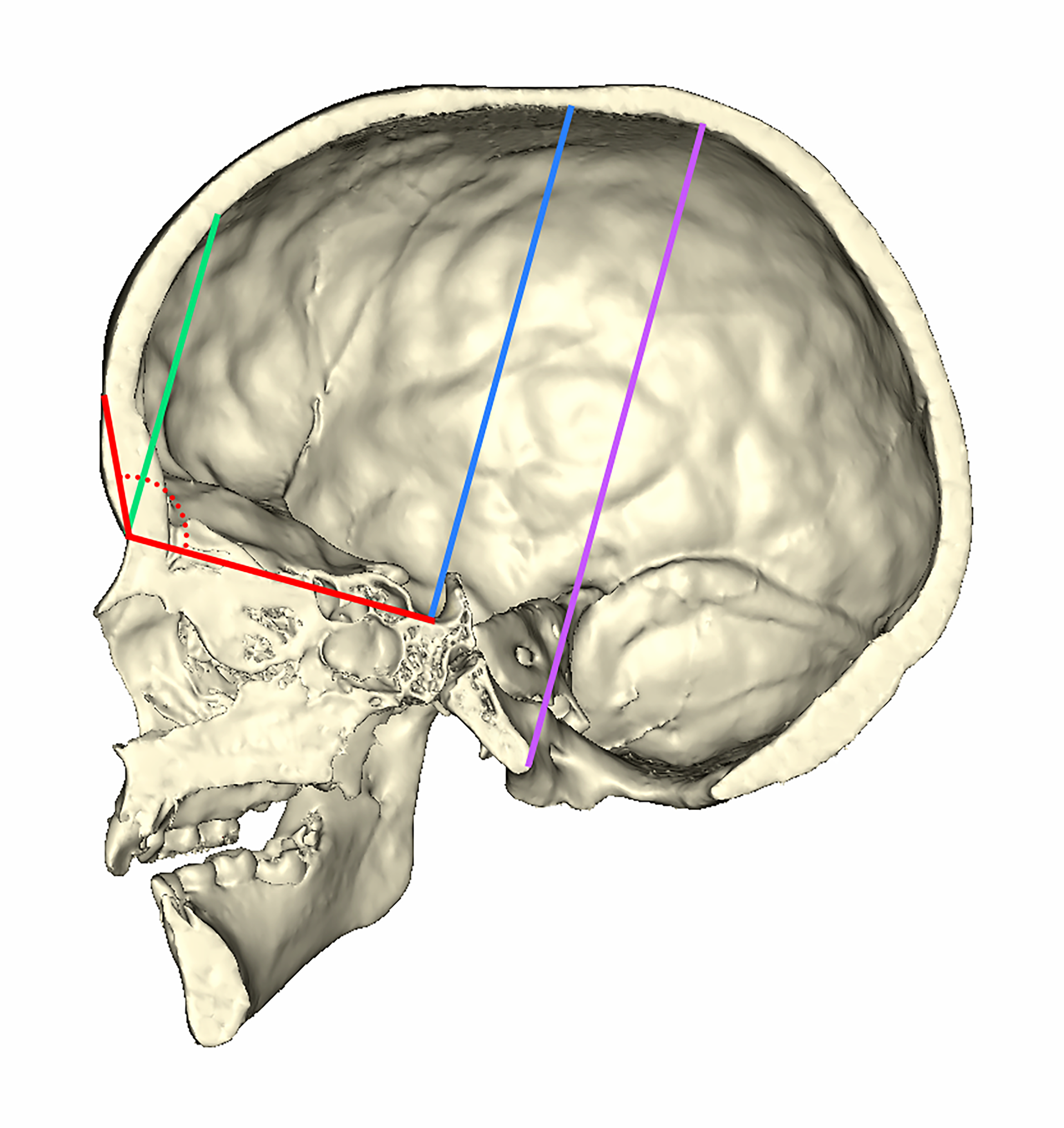

Supplement: Supplementary file 1 — Supplementary file1 (TIF 28700 KB) [file 381_2024_6465_MOESM1_ESM.tif]

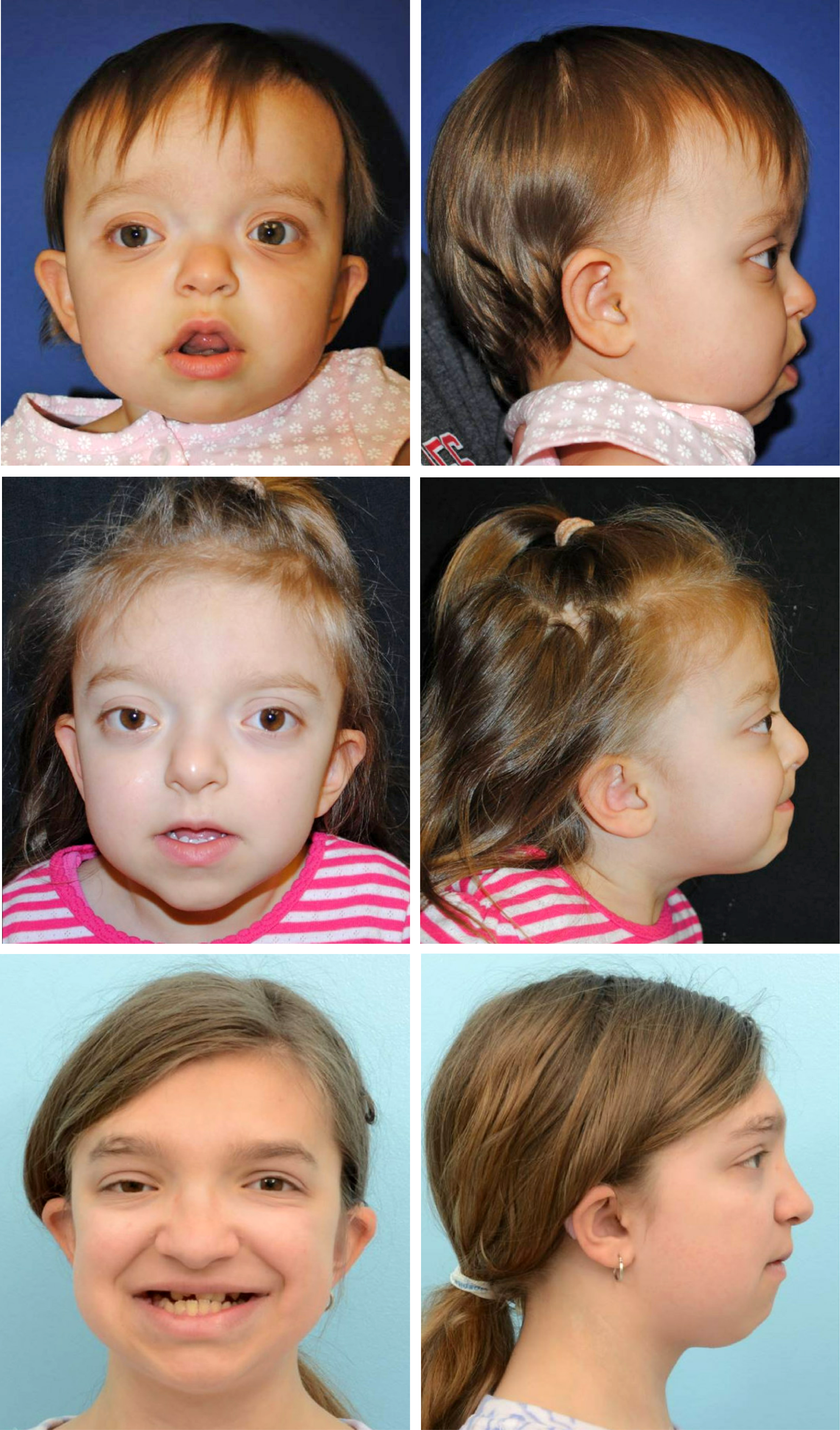

Supplement: Supplementary file 2 — Supplementary file2 (PNG 4280 KB) [file 381_2024_6465_MOESM2_ESM.png]
