# Supplementary material for: Early posterior vault distraction osteogenesis changes the syndromic craniosynostosis treatment paradigm: long-term outcomes of a 23-year cohort study
Source: Childs Nerv Syst. 2024 Jun 21;40(9):2811–23. doi: 10.1007/s00381-024-06465-x (PMC11322207; doi:10.1007/s00381-024-06465-x)
Supplement: Supplementary file 3 — Supplementary file3 (DOCX 14.6 KB) [file 381_2024_6465_MOESM3_ESM.docx]

| Supplemental Table 1. Surgical Interventions in the First Eight Years of Life Among Patients Presenting Within One Year of Age (n=46). | | | |
| --- | --- | --- | --- |
|  | **PVDO**  **Cohort** (%) | **Conventional Cohort** (%) | ***p*** |
| No. of patients | 26 (57) | 20 (43) |  |
| Mean age at presentation, yr | 0.2 ± 0.2 | 0.2 ± 0.3 | 0.456 |
| Mean age at initial vault surgery, yr | 0.9 ± 0.7 | 1.2 ± 2.0 | 0.429 |
| Mean age at primary FOA, yr | 2.3 ± 1.6 | 1.0 ± 0.7 | **<0.001** |
| Mean age at secondary FOA, yr | 8.7 | 7.6 ± 3.3 |  |
| Mean no. of FOAs  0  1  2 | 0.7 ± 0.5  8 (31)  18 (69)  0 (0) | 1.2 ± 0.7  0 (0)  14 (70)  5 (30) | **0.002** |
| Mean no. of major craniofacial interventions  1  2  3  4  5  6 | 2.7 ± 1.0  1 (4)  12 (46)  8 (31)  4 (15)  0 (0)  1 (4) | 2.2 ± 1.4  9 (45)  3 (12)  3 (12)  2 (8)  2 (8)  0 (0) | 0.079 |
| *PVDO*, posterior vault distraction osteogenesis; *FOA*, fronto-orbital advancement. | | | |
