# Supplementary material for: Early posterior vault distraction osteogenesis changes the syndromic craniosynostosis treatment paradigm: long-term outcomes of a 23-year cohort study
Source: Childs Nerv Syst. 2024 Jun 21;40(9):2811–23. doi: 10.1007/s00381-024-06465-x (PMC11322207; doi:10.1007/s00381-024-06465-x)
Supplement: Supplementary file 4 — Supplementary file4 (DOCX 13.8 KB) [file 381_2024_6465_MOESM4_ESM.docx]

| Supplemental Table 2. Preoperative Craniometrics in PVDO and Conventional Cohorts (n=30). | | | |
| --- | --- | --- | --- |
|  | **PVDO Cohort**  (n=20) | **Conventional Cohort**  (n=10) | ***p*** |
| Anterior Cranial Height, mm | 81.2 ± 17.2 | 76.9 ± 14.9 | 0.681 |
| Middle Cranial Height, mm | 101.1 ± 10.8 | 100.8 ± 10.8 | 0.895 |
| Posterior Cranial Height, mm | 107.9 ± 13.9 | 111.4 ± 16.9 | 0.468 |
| Cranial Width, mm | 115.8 ± 13.7 | 114.5 ± 18.2 | 0.947 |
| Cranial Length, mm | 120.6 ± 21.3 | 126.0 ± 24.2 | 0.475 |
| Frontal bossing angle,° | 121.9 ± 9.5 | 123.0 ± 10.6 | 0.779 |
| Turricephaly Index | 1.29 ± 0.20 | 1.27 ± 0.19 | 0.779 |
| *PVDO*, posterior vault distraction osteogenesis; *mm*, millimeters. | | | |
