# Supplementary material for: Early posterior vault distraction osteogenesis changes the syndromic craniosynostosis treatment paradigm: long-term outcomes of a 23-year cohort study
Source: Childs Nerv Syst. 2024 Jun 21;40(9):2811–23. doi: 10.1007/s00381-024-06465-x (PMC11322207; doi:10.1007/s00381-024-06465-x)
Supplement: Supplementary file 5 — Supplementary file5 (DOCX 14.6 KB) [file 381_2024_6465_MOESM5_ESM.docx]

| Supplemental Table 3. Preoperative Craniometrics by Syndrome Type (n=30). | | | | | | |
| --- | --- | --- | --- | --- | --- | --- |
|  | **Apert**  (n=9) | **Crouzon**  (n=9) | **Pfeiffer**  (n=3) | **Muenke**  (n=5) | **Saethre-Chotzen** (n=4) | ***p*** |
| Anterior Cranial Height, mm | 93.7 ± 9.8 | 62.4 ± 8.8 | 74.7 ± 20.7 | 81.7 ± 4.3 | 89.1 ± 13.3 | **<0.001** |
| Middle Cranial Height, mm | 107.1 ± 14.3 | 98.6 ± 7.7 | 99.5 ± 6.3 | 97.2 ± 3.8 | 98.9 ± 13.5 | 0.383 |
| Posterior Cranial Height, mm | 109.4 ± 19.7 | 117.9 ± 10.2 | 113.7 ± 4.3 | 107.9 ± 13.9 | 96.4 ± 13.2 | 0.069 |
| Cranial Width, mm | 115.9 ± 19.9 | 123.5 ± 11.2 | 110.9 ± 8.5 | 111.0 ± 8.6 | 104.6 ± 15.6 | 0.252 |
| Cranial Length, mm | 116.2 ± 19.5 | 141.6 ± 23.5 | 124.5 ± 14.6 | 108.9 ± 9.8 | 108.4 ± 11.6 | **0.014** |
| Frontal bossing angle,° | 129.0 ± 8.3 | 114.6 ± 6.9 | 116.3 ± 15.9 | 125.6 ± 2.9 | 124.8 ± 7.3 | **0.009** |
| Turricephaly Index | 1.17 ± 0.13 | 1.46 ± 0.22 | 1.26 ± 0.22 | 1.25 ± 0.07 | 1.17 ± 0.08 | **0.007** |
| *PVDO*, posterior vault distraction osteogenesis; *mm*, millimeters. | | | | | | |
